# An exploratory study on the association between vitamin D intake or synthesis and sleep health in young adult women: an observational study

**DOI:** 10.1186/s40101-026-00436-8

**Published:** 2026-07-16

**Authors:** Rika Yoshizu, Toru Sasaki, Hideaki Nakajima, Hiromi Eto, Takuya Shuo, Hideaki Kondo

**Affiliations:** 1 Department of Reproductive Health, Health Sciences, Graduate School of Biomedical Sciences, Nagasaki University, 1-12-4, Sakamoto, Nagasaki-City, Nagasaki 852-8523 Japan; 2 National Institute for Environmental Studies, 16-2 Onogawa, Tsukuba, Ibaraki 305-8506 Japan; 3 Department of Reproductive Health, Institute of Biomedical Sciences, Nagasaki University, 1-7-1 Sakamoto, Nagasaki-City, 852-8520 Japan; 4 Faculty of Health and Medical Sciences, Hokuriku University, 1-1 Taiyogaoka, Kanazawa, 920-1180 Japan; 5 Department of General Medicine, Nagasaki University Hospital, 1-7-1, Nagasaki-City, 852-8501 Japan

**Keywords:** Depression, Diet history questionnaire, Insomnia, Sleep health, Ultraviolet, Vitamin D, Vitamin D_3_ synthesis, 25-Hydroxyvitamin D

## Abstract

**Background:**

Vitamin D deficiency, which is highly prevalent in modern society, is associated with health issues, including sleep–wake disorders and depression. This pilot study aimed to examine the relationship between dietary vitamin D intake or vitamin D synthesis from ultraviolet exposure and serum 25-hydroxyvitamin D (25[OH]D) levels as an indicator of vitamin D status, as well as their association with sleep health and depressive symptoms.

**Methods:**

Twenty-nine young adult women participated in two surveys conducted in spring and summer. Vitamin D intake was estimated based on responses to a dietary questionnaire. The amount of vitamin D synthesized in the skin through ultraviolet exposure was calculated by estimating ultraviolet exposure over a 2-week period. Serum 25(OH)D concentrations were measured using liquid chromatography-tandem mass spectrometry. Sleep habits were assessed using a 2-week sleep diary, and depression and insomnia symptoms were evaluated using questionnaires.

**Results:**

The mean (SD) serum 25(OH)D concentrations in spring and summer were 8.9 (2.9) and 15.5 (4.8) ng/mL, respectively. The median (IQR) dietary vitamin D intake adjusted for energy expenditure based on dietary surveys was 8.5 (6.6, 9.2) μg/day in spring and 6.2 (4.3, 10.2) μg/day in summer (*P* = 0.40). The median (IQR) amount of vitamin D produced from ultraviolet exposure increased from 10.5 (4.6, 17.4) μg/day in spring to 17.0 (10.1, 34.5) μg/day in summer (*P* = 0.009). Among those with low serum 25(OH)D concentrations or low vitamin D intake in summer, the delayed midpoint of sleep was observed. No significant correlation was found between changes in serum 25(OH)D concentrations from spring to summer and changes in depression or insomnia symptoms.

**Conclusions:**

All participants exhibited vitamin D deficiency during the spring term. In contrast, during the summer term, serum 25(OH)D levels increased, primarily due to enhanced vitamin D synthesis resulting from ultraviolet exposure of the skin. However, the observed increase in serum 25(OH)D levels from spring to summer did not correlate with any improvement in depression or insomnia symptoms.

**Supplementary Information:**

The online version contains supplementary material available at https://doi.org/10.1186/s40101-026-00436-8.

## Introduction

Vitamin D in the human body is categorized into vitamin D_3_, which is synthesized in the skin and partially sourced from animal-based foods, and plant-derived vitamin D_2_ [1]. Both forms undergo hydroxylation at the 25th position in the liver to become 25-hydroxyvitamin D (25[OH]D), and further hydroxylation at the 1α position in the kidneys synthesizes the highly biologically active 1,25-dihydroxyvitamin D (1,25[OH]2D). Because 1,25(OH)2D is rapidly synthesized and fluctuates in response to enzyme induction during vitamin D deficiency, 25(OH)D, which has a half-life of 2–3 weeks [2, 3], is commonly used as an indicator of deficiency. The criteria for vitamin D deficiency differ across countries and organizations; however, a 25(OH)D level below 20 ng/mL is generally recognized as deficient, whereas levels below 10–12 ng/mL are considered indicative of severe deficiency [4, 5].

Vitamin D deficiency is a significant concern in modern society. A comprehensive meta-analysis that reviewed 308 epidemiological studies involving 7.95 million participants from 81 countries and published between 2000 and 2022 found that the prevalence of vitamin D deficiency was 47.9% (95% confidence interval (CI), 44.9–50.9%). In areas located between latitudes 20° and 40° north, such as Japan, the prevalence was notably higher at 60.2% (55.5–64.9%), with this trend consistent across various age groups [6]. A report focusing on health checkup participants in Japan for the 2019 fiscal year (*n* = 5481) indicated an even higher prevalence of 79% [7]. Our research involving pregnant women (*n* = 203) and female university students (*n* = 224) revealed deficiency rates of 79.8% and 80.8%, respectively [8, 9]. The widespread nature of vitamin D deficiency is being rigorously examined as a public health issue that impacts individuals of all ages.

The association between vitamin D deficiency and physical and mental disorders is widely recognized. Vitamin D deficiency increases the risk of obesity [10, 11], metabolic syndrome [12, 13], type 2 diabetes [14, 15], atrial fibrillation [16], cardiovascular diseases [17], as well as breast and thyroid cancers [18, 19]. In patients with type 2 diabetes, vitamin D deficiency or insufficiency also increases the risk of all-cause mortality and mortality due to cardiovascular diseases [20]. Furthermore, it is associated with dementia [21] and depression [22–24], and severe vitamin D deficiency has been linked to treatment-resistant psychiatric symptoms [22]. Although numerous vitamin D supplementation therapies have been implemented for various diseases and conditions and do not necessarily contribute to improvements in all outcomes, many reports have demonstrated their efficacy and benefits [5, 25, 26].

Addressing vitamin D deficiency requires prioritizing the enhancement of dietary vitamin D intake and the promotion of natural vitamin D synthesis in the skin before considering supplementation or active vitamin D prescriptions. Enhancing dietary vitamin D intake necessitates an understanding of the vitamin D content in consumed foods and, where feasible, efforts to augment this content, such as through exposure to ultraviolet (UV) radiation [10]. To facilitate the cutaneous synthesis of vitamin D via UV exposure, it is imperative to ensure sufficient exposure to sunlight, both in terms of duration and intensity, while mitigating the risk of adverse effects from excessive UV radiation [11, 12]. Ultimately, it is crucial to evaluate whether increased intake and synthesis of vitamin D are reflected by elevated blood 25(OH)D levels and whether these changes contribute to the amelioration of health conditions.

In addressing the multifaceted challenges associated with vitamin D deficiency, it is crucial to consider not only factors such as diet and solar exposure during waking hours but also the role of sleep. Vitamin D deficiency has been linked to short sleep duration, reduced sleep quality, obstructive sleep apnea [13], and restless legs syndrome (RLS) [8, 14]. In addition, severe vitamin D deficiency has been associated with the onset of RLS and excessive daytime sleepiness, and there have been reports of cases in which vitamin D supplementation improved these symptoms [15, 16]. Among individuals with low levels of physical activity, many suffer from vitamin D deficiency [17]; insomnia symptoms, social jet lag [9], and daytime sleepiness [18] may be underlying factors. It is possible that sleep–wake issues and vitamin D deficiency influence each other.

This study aimed to elucidate the extent to which cutaneous vitamin D synthesis, as estimated from UV exposure, contributes to the elevation of serum 25(OH)D levels during summer months under typical living conditions. The research focused on young adult women and included evaluations of dietary vitamin D intake, with surveys conducted twice, once in spring and once in summer. Additionally, as a pilot study, it explored whether an increase in serum 25(OH)D levels during summer is associated with improvements in depression and insomnia symptoms. Furthermore, this study investigated the interrelationships among serum 25(OH)D levels, vitamin D synthesis, vitamin D intake, and lifestyle habits, including sleep–wake patterns.

## Methods

### Participants

The study involved female university and graduate students (*n* = 29). This study was conducted in an exploratory manner. Owing to the absence of existing reports—at least within the scope of our search—regarding the extent to which vitamin D synthesis through ultraviolet exposure in typical living environments influences symptoms of depression or insomnia, a formal calculation of the sample size was not undertaken. Ethical approval was obtained from the Ethics Committee of the Graduate School of Biomedical Sciences, Nagasaki University (approval no.: 24111401). Participants were informed about the study verbally and in writing, and informed consent was secured. Exclusion criteria were liver cirrhosis, chronic kidney disease stage G3 or higher (eGFR < 60 mL/min/m^1.73^), a history of intestinal resection, pregnancy or breastfeeding, photosensitivity, and any conditions deemed unsuitable by the researchers. Individuals who had been taking vitamin D-containing supplements were eligible to participate 30 days after discontinuing their use.

### Survey procedure

The survey was conducted twice: once in the spring, when ultraviolet radiation is low, and once in the summer, when it is higher. The spring survey was conducted from March 11 to April 16, 2025, and the summer survey was conducted from July 22 to September 16, 2025. During 2 weeks in each period, in addition to keeping a sleep diary, participants also noted the regions and times they spent outdoors during the day. When going outside, they recorded whether they used a parasol, whether they applied sunscreen, and which areas of skin were exposed. At the end of the 2-week survey period, participants were asked to complete questionnaires regarding background factors, dietary habits, depressive symptoms, and insomnia symptoms. For background factors, information was collected on height, weight, smoking habits, drinking habits, comorbidities, premenstrual syndrome, and exercise habits. In addition, 15 mL of whole blood was collected on the first day of the survey and again 2 weeks later at the end of the survey. Serum 25(OH)D concentrations were measured using liquid chromatography-tandem mass spectrometry (LC-MS/MS) and intact parathyroid hormone (PTH) levels were measured using an electrochemiluminescence immunoassay (see Supplementary Methods). Intact PTH was measured because high vitamin D deficiency tends to cause secondary elevation of PTH levels, even when blood calcium levels are normal.

### Dietary intake survey

Vitamin D intake from diet was evaluated using a brief self-administered diet history questionnaire (BDHQ). This questionnaire was developed based on the self-administered diet history questionnaire, which estimates the intake of 151 types of foods and more than 100 types of nutrients based on dietary habits over the past month. The BDHQ calculates the intake of 58 types of foods and more than 100 types of nutrients [13, 14]. The time required to complete the questionnaire is approximately 15 min. In this study, participants were asked to complete the questionnaire based on their dietary habits during the 2 weeks prior to the date of response. The validity of using this method to assess vitamin D intake, with 25(OH)D as an indicator, has also been reported [15, 16].

The dietary survey revealed that some individuals reported an obviously insufficient energy intake, with actual food intake being significantly underreported. Because vitamin D intake correlates with energy intake (Supplementary Fig. 1), vitamin D intake was adjusted based on the estimated energy intake (adjusted vitamin D intake). When body weight is relatively stable, energy intake is almost equal to energy expenditure; therefore, participants’ energy intake was estimated using the value of 38 kcal/kg/day for energy expenditure per kilogram of body weight, as reported in a systematic review of Japanese adult women [17]. According to the Japanese Dietary Reference Intakes (2025 edition), the recommended vitamin D intake for adults is 9.0 μg/day [18]. In this study, we calculated the proportion of individuals whose intake was below this recommended amount. We also calculated vitamin D intake per 1000 kcal of energy intake using the density method.

### Vitamin D synthesis from estimated ultraviolet exposure

The estimated UV-B exposure was calculated based on 2 weeks of activity records. The area of skin exposed was determined by calculating each participant’s body surface area using an estimation formula for Japanese adult females and applying the proportion of each body part's surface area [19]. The upper arm and forearm were each evenly divided into proximal and distal sections. Owing to the effect of hair covering certain parts of the skin and the fact that the side opposite to direct sunlight is not exposed, the percentage of UV-B exposure area for each body part was calculated as follows: 25% for the head, 0% for the ears, and 50% for the neck, upper arms, forearms, hands, thighs, lower legs, and feet. Areas covered by sunscreen were excluded from the exposed area. Additionally, when a parasol was used, the skin exposure area was multiplied by 0.7.

The amount of vitamin D synthesized by UV-B radiation was estimated from the hourly global solar radiation data published by the Japan Meteorological Agency for the Nagasaki Meteorological Observatory and the nearest meteorological observatories in each prefecture, using the UV-B radiation levels for the relevant time period on the relevant days. The amount of vitamin D synthesized was calculated by multiplying the vitamin D production rate by the participant's skin type, exposed body surface area, exposure time, and the amount of vitamin D synthesizing UV-B radiation. Depending on the participant's Fitzpatrick skin type, an adjustment factor was applied to the UV-B radiation: 1.0 for skin type I, 0.8 for type II, 0.67 for type III, and 0.5 for type IV [20]. From the amount of vitamin D synthesis during each period of outdoor activity, the average daily production over 2 weeks was also calculated [16, 21]. Days spent abroad during the survey period were excluded, and the amount synthesized per day spent in Japan was calculated.

### Questionnaires

Depressive symptoms were assessed using the Patient Health Questionnaire-9 (PHQ-9). This questionnaire consists of nine items, with a total score ranging from 0 to 27; higher scores indicate more severe depressive symptoms. Both the sensitivity and specificity for major depression at a total score of 10 or above have been reported to be 88%. Scores of 5–9, 10–14, 15–19, and 20–27 are classified as mild, moderate, severe, and very severe, respectively [22]. In this study, participants were divided into two groups: those with a total score of 5 or higher and those with a score below 5.

Insomnia symptoms were assessed using the Athens Insomnia Scale. This questionnaire consists of eight items, with a total score ranging from 0 to 24 points. Higher scores indicate more severe insomnia symptoms. Scores of 6–9, 10–15, and 16–24 are classified as mild, moderate, and severe, respectively [23–25]. The total scores were divided into two groups: those with scores of ≥ 6 and those with scores < 6. Difficulty falling asleep, difficulty maintaining sleep, early morning awakening, and daytime sleepiness were each categorized into two groups: those who experienced the symptom and those who did not.

RLS was identified using a single-question survey: “When you try to relax in the evening or sleep at night, do you ever have unpleasant, restless feelings in your legs that can be relieved by walking or movement?” [26]. Respondents answered either “yes” or “no.” Participants who answered “yes” were categorized as the RLS group, and all others were categorized as the non-RLS group.

### Sleep diary

Sleep patterns were assessed using sleep diaries over a 2-week period. Each day, bedtime, sleep latency, wake after sleep onset, final awakening time, and wake-up time were recorded, and from these, sleep onset time, time in bed, sleep duration, midpoint of sleep, and sleep efficiency were calculated. For each variable, the minimum, first quartile, median, third quartile, maximum, range, and interquartile range over the 2-week period were calculated. There were instances in which the duration of nighttime sleep was 0 h; in those cases, both time in bed and nighttime sleep duration were calculated as 0 h. The irregularity of total sleep time and sleep midpoint was evaluated by calculating the sum of the absolute differences between the median value over 2 weeks and the daily data, and then converting this to a value per week. Days spent overseas were excluded, and values were adjusted to a per-week basis. The sense of restfulness upon waking was assessed daily using a 6-point scale, with “not at all” scored as 0 and “very well rested” scored as 5. Similarly, daytime impairment was assessed using a 6-point scale, with “not at all” scored as 0 and “very much” scored as 5. The total scores for the 2-week period ranged from 0 to 70 for each category, which were then converted to a 0–100 scale.

### Statistics

Statistical analyses were performed using R ver. 4.5.2 and EZR ver. 1.708. Continuous variables with a normal distribution are presented as the mean (standard deviation: SD), whereas those without a normal distribution are presented as the median (interquartile range: IQR). The significance level was set at 0.05. Pearson’s correlation coefficient was used when normality was confirmed, and Spearman’s rank correlation coefficient was used when normality was not observed for the correlation coefficients. For comparisons between two independent groups of continuous variables, an unpaired *t*-test was used for variables with a normal distribution, and the Mann–Whitney *U* test for those without. For comparisons between two paired groups of continuous variables, a paired *t*-test was used when normality was confirmed, and the Wilcoxon rank sum test when it was not. Fisher’s exact test was used to test the independence of nominal variables in independent groups. McNemar’s test was employed to compare the proportions of paired nominal variables.

## Results

The median age (IQR) of the women studied (*n* = 29) was 23.0 (21.0, 24.0) years. Skin type III accounted for nearly half, that is, 48.3% (Table 1). None of the participants were engaged in shift work during the survey period. In the summer term, one participant was absent; therefore, the comparison between the spring and summer terms was conducted with 28 participants. In the spring survey, two individuals traveled abroad for 3 and 6 days during the survey period. These days were excluded from the estimation of vitamin D synthesis and the calculation of sleep–wake parameters, and the weekly parameters were adjusted accordingly.

**Table 1:** Demographic characteristics

*n*	29
Age, median (IQR)	23.0 (21.0, 24.0)
BMI kg/m^2^, median (IQR)	20.6 (19.0, 21.9)
≥ 25 kg/m^2^, *n* (%)	1 (3.4)
Skin type	
Ⅰ, *n* (%)	2 (6.9)
Ⅱ, *n* (%)	8 (27.6)
Ⅲ, *n* (%)	14 (48.3)
Ⅳ, *n* (%)	5 (17.2)
Living alone, *n* (%)	21 (72.4)
Loving with parents, *n* (%)	7 (24.1)
Non-smoking, *n* (%)	29 (100.0)
Regular exercise, *n* (%)	14 (48.3)

*BMI *body mass index, *IQR *interquartile range

### Serum 25(OH)D concentrations in the spring and summer seasons

**Fig. 1:**
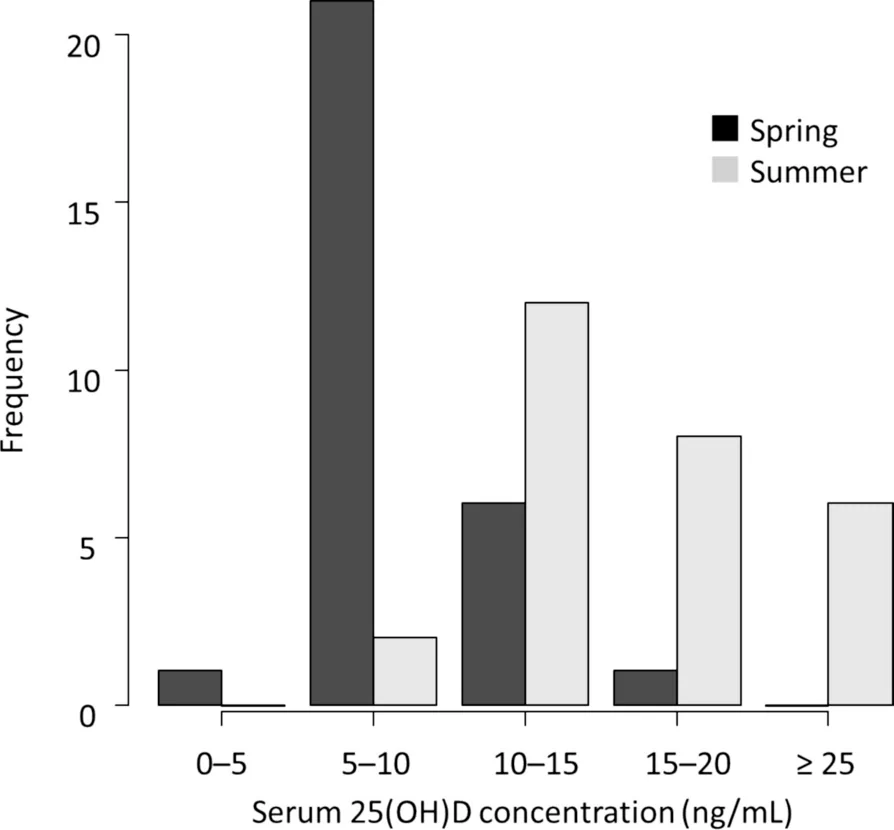
Histogram of serum 25(OH)D concentrations during spring and summer. The frequency is presented for each 5 ng/mL increment of serum 25(OH)D concentration. During the spring season, all participants exhibited a deficient state with levels below 20 ng/mL, and 22 individuals (75.9%) demonstrated severe deficiency with levels below 10 ng/mL. Notably, even in the summer, a substantial proportion (78.6%) remained deficient with levels below 20 ng/mL.25(OH)D, 25-hydroxyvitamin D

**Fig. 2:**
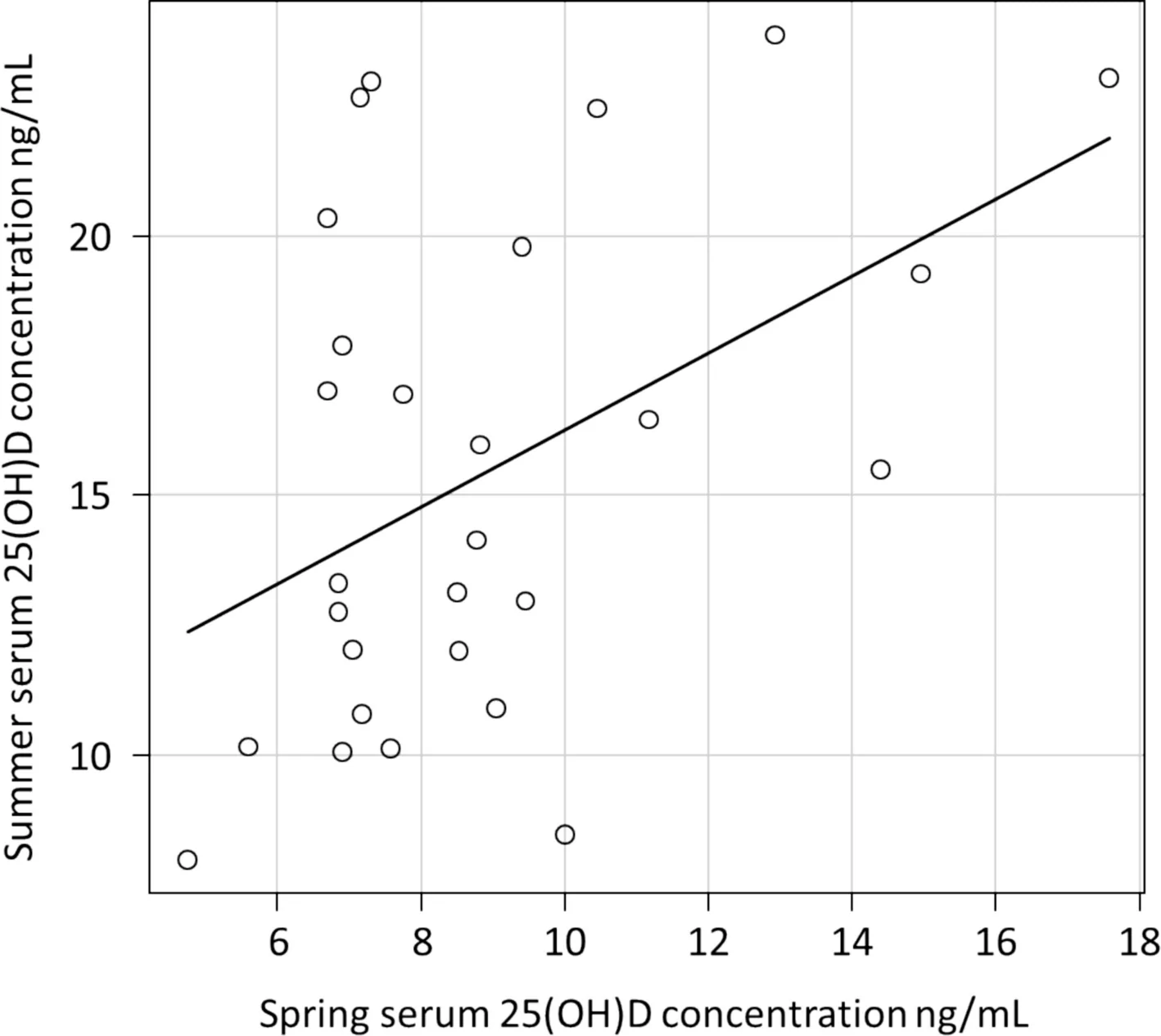
Scatter plot of serum 25(OH)D concentrations during spring and summer. There was a moderate correlation between the two, with a Pearson correlation coefficient (95% CI) of 0.45 (0.10, 0.71) (*P* = 0.02). 25(OH)D, 25-hydroxyvitamin D

In this study population, 25(OH)D_2_ was undetectable; all measurements were for 25(OH)D_3_. The mean serum 25(OH)D concentrations (SD) in spring and summer were 8.9 (2.9) and 15.5 (4.8) ng/mL, respectively, with significantly higher values in summer than in spring (paired *t*-test, *P* < 0.001). In spring, all participants were in a deficient state with levels below 20 ng/mL, and 22 individuals (75.9%) had severe deficiency with levels below 10 ng/mL. Even in summer, the proportion of individuals deficient in 25(OH)D < 20 ng/mL remained high at 78.6% (Fig. 1). The difference between the two seasons (95% confidence interval (CI)) was 6.6 (4.9, 8.3). The two measurements showed a moderate correlation, with Pearson’s correlation coefficient (95% CI) of 0.45 (0.10, 0.71) (*P* = 0.02, Fig. 2). No significant correlation was observed between serum 25(OH)D concentration and serum intact PTH concentration (Supplementary Fig. 2).

**Fig. 3:**
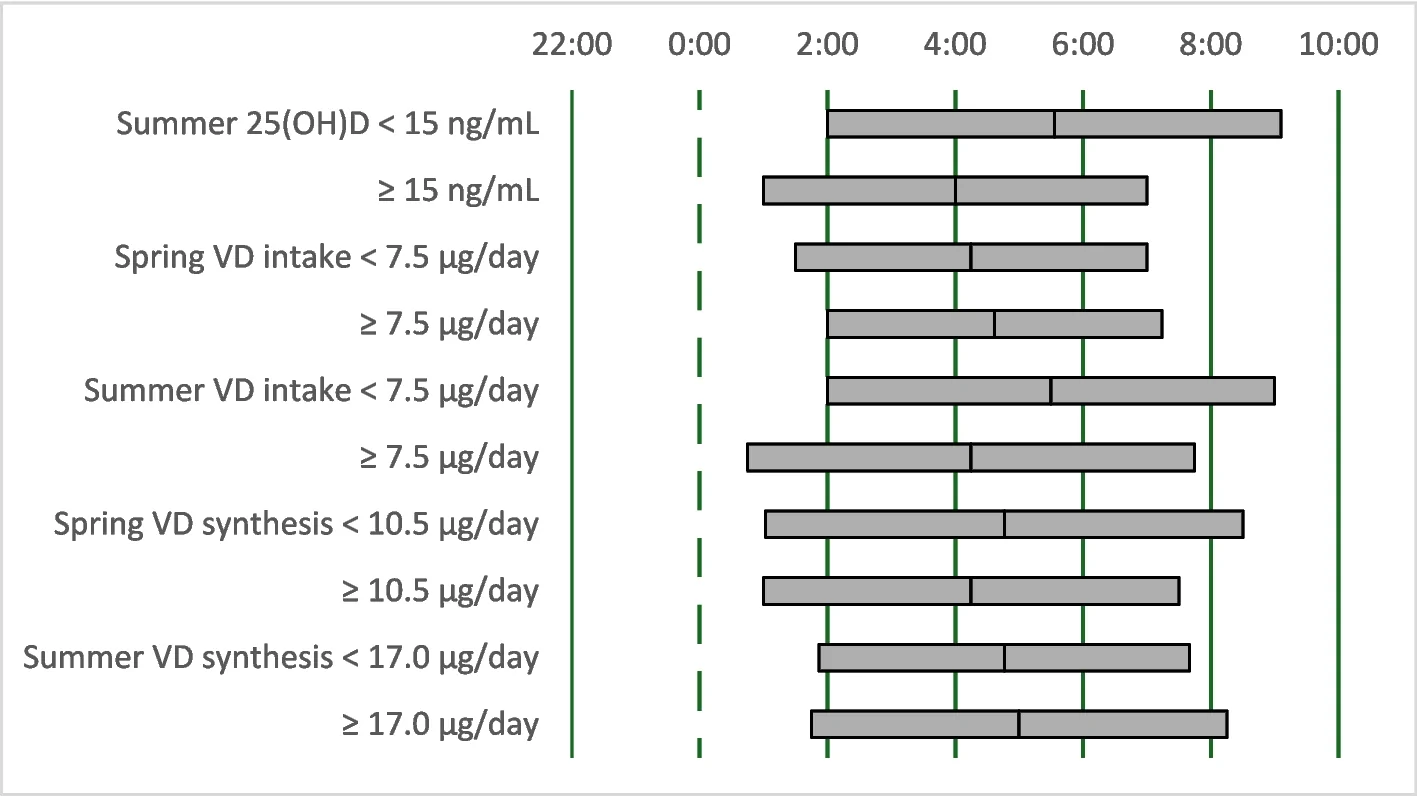
Sleep phases in each of the two groups for the vitamin D parameters. Serum 25(OH)D concentration, vitamin D intake, and vitamin D synthesis amount are each divided into two groups and illustrated using the median values from bedtime to final awakening time for each group. The central bar represents the midpoint of sleep. 25(OH)D, 25-hydroxyvitamin D; VD, vitamin D

The participants were divided into two groups based on the median serum 25(OH)D concentration, and a comparison was made between the high- and low-25 (OH) D groups. However, no significant findings were observed in the spring. In the summer, compared to the group with 25(OH)D ≥ 15 ng/mL, the group with 25(OH)D < 15 ng/mL showed the delayed midpoint of sleep and later wake-up times (Fig. 3), and demonstrated greater irregularity in sleep duration. Although not statistically significant, both vitamin D intake and synthesis tended to be lower in the group with 25(OH)D < 15 ng/mL (Supplementary Table 1).

### Increase in serum 25(OH)D concentrations from spring to summer and vitamin D intake and synthesis

Serum 25(OH)D concentrations increased in all but one individual during the summer compared to those in spring. Scatter plots of the changes in serum 25(OH)D concentrations versus changes in dietary vitamin D intake and changes in vitamin D synthesis from skin exposure to UV-B radiation showed no significant correlations, with Spearman’s *ρ* values of 0.09 (*P* = 0.66) and 0.21 (*P* = 0.28), respectively (Supplementary Fig. 3). Similar findings were observed when the rate of increase from spring to summer was re-evaluated, not just the amount of change. However, the rate of increase (IQR) from spring to summer was significantly higher for vitamin D synthesis in the skin, that is, − 23.4% (− 39.8%, 23.8%) for dietary intake and 89.5% (− 13.5%, 168.8%) for skin synthesis, respectively (Wilcoxon rank sum test, *P* < 0.001).

### Vitamin D intake from food

**Fig. 4:**
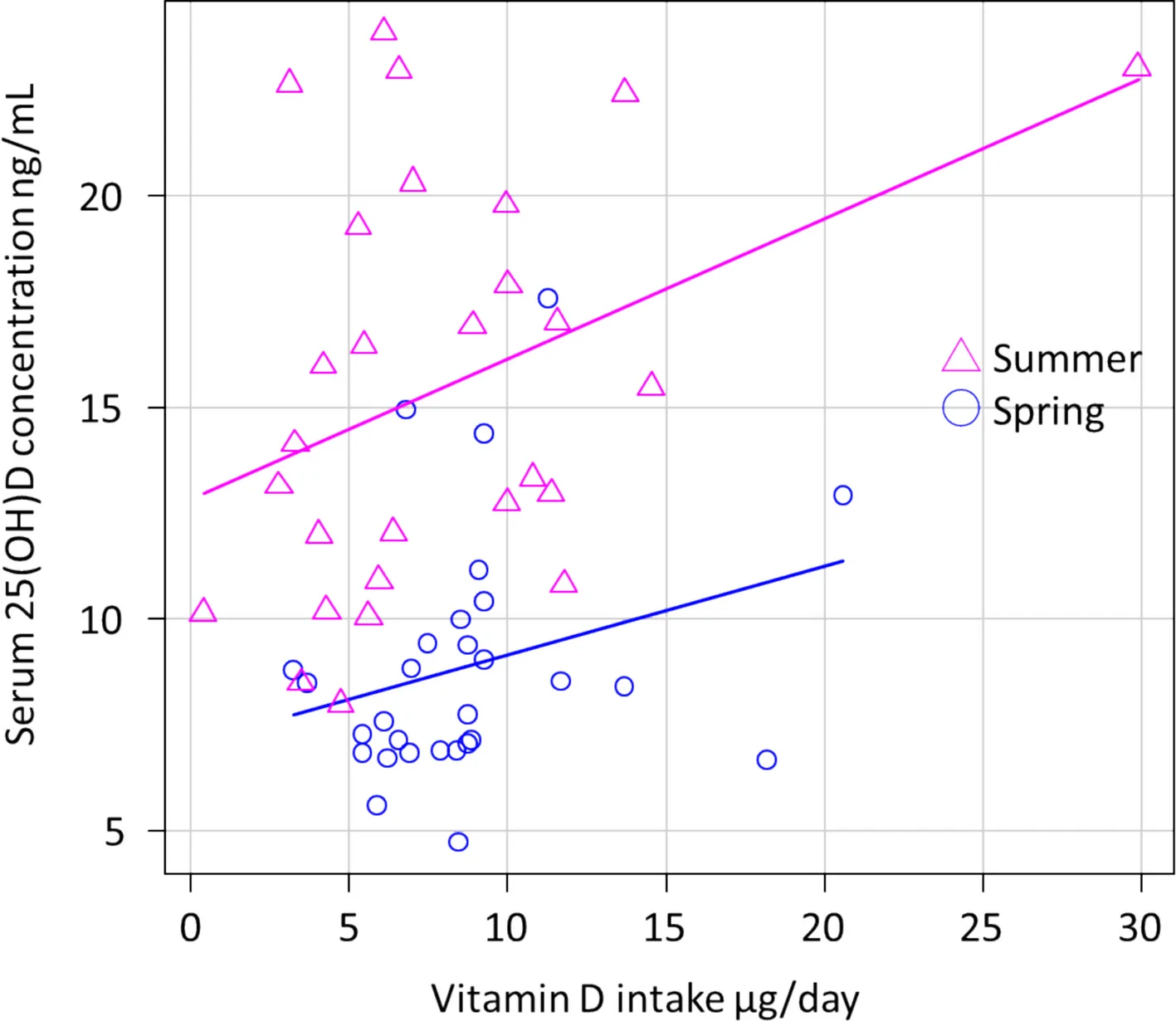
Scatter plot of vitamin D intake and serum 25(OH)D concentration. The scatter plot shows the relationship between vitamin D intake adjusted for energy consumption based on dietary surveys and serum 25(OH)D concentrations separated by spring (○) and summer (△). The Spearman rank correlation coefficients for spring and summer were *ρ* = 0.37 (*P* = 0.05) and *ρ* = 0.35 (*P* = 0.07), respectively. 25(OH)D, 25-hydroxyvitamin D

The median (IQR) vitamin D intake adjusted for energy consumption based on dietary surveys was 8.5 (6.6, 9.2) μg/day in spring and 6.2 (4.3, 10.2) μg/day in summer, with no significant difference observed (Wilcoxon rank sum test, *P* = 0.40). The Spearman rank correlation coefficient between vitamin D intake and serum 25(OH)D concentration was *ρ* = 0.37 (*P* = 0.05) and *ρ* = 0.35 (*P* = 0.07), respectively (Fig. 4). Adjusted vitamin D intake showed a significant positive correlation with protein intake in summer (Spearman’s *ρ* = 0.61, *P* < 0.001) and a significant positive correlation was also found between the increase in vitamin D intake and the increase in protein intake from spring to summer (Spearman’s *ρ* = 0.38, *P* = 0.04, Table 2). No significant association was found between the increase in serum 25(OH)D concentration and the increase in vitamin D intake from spring to summer (Spearman’s *ρ* = 0.21, *P* = 0.28).

**Table 2:** Correlation between vitamin D and individual nutrients intake

	Total	Spring	Summer	Amount of increased VD intake
Carbohydrate	− 0.27	− 0.25	− 0.30	− 0.14
Protein	0.54*	0.36	0.61*	0.38**
Fat	0.14	0.00	0.18	0.04

The Spearman rank correlation coefficient is shown for vitamin D intake adjusted for energy consumption and the proportion of each energy-producing nutrient in the total energy intake. The correlation between the increase in vitamin D intake from spring to summer and changes in energy and nutrient intake is also presented. * *P* < 0.001, ** *P* = 0.04. VD, vitamin D

Since there were no significant seasonal variations in vitamin D intake during the spring and summer periods, the participants were divided into two groups based on the average of the median-adjusted vitamin D intake for spring and summer, which was 7.5 μg/day, to explore factors associated with vitamin D intake in these seasons. In spring, no significant findings were observed in the group with a vitamin D intake of ≥ 7.5 μg/day compared to the group with < 7.5 μg/day, except for a higher protein intake (Supplementary Table 2). In summer, the group with vitamin D intake ≥ 7.5 μg/day had a higher protein intake and a lower carbohydrate intake, exhibited advanced sleep phase while total sleep duration remained unchanged (Fig. 3), and had a higher proportion of individuals eating breakfast (Table 3 and Supplementary Table 3).

**Table 3:** Comparison of sleep–wake patterns and percentage breakfast intake between two groups with summer vitamin D intake of < 7.5 μg/day and ≥ 7.5 μg/day

	VD intake < 7.5 μg/day	VD intake ≥ 7.5 μg/day	*P* value
*n*	17	11	
TST h, median (IQR)	6.00 (5.75, 7.00)	6.00 (5.75, 7.00)	0.87
< 6 h, *n* (%)	2 (11.8)	1 (9.1)	1.00
6–7 h. *n* (%)	7 (41.2)	5 (45.5)	
≥ 7 h, *n* (%)	8 (47.1)	5 (45.5)	
Range of TST hr, median (IQR)	5.00 (2.75, 8.50)	3.00 (2.75, 4.00)	0.10
Irregularity of TST hr/week, median (IQR)	6.75 (4.96, 10.75)	5.00 (4.50, 7.00)	0.23
Bedtime h:mm, median (IQR)	1:45 (1:00, 2:30)	0:30 (0:00, 1:22)	0.04
Sleep latency min, median (IQR)	30.0 (0.0, 30.0)	22.5 (15.0, 30.0)	0.69
Sleep onset h:mm, median (IQR)	2:00 (1:30, 2:45)	0:45 (0:22, 1:45)	0.04
MS h:mm, median (IQR)	5:30 (4:45, 6:00)	4:15 (3:41, 4:31)	0.01
Irregularity of MS min/week, median (IQR)	326.3 (262.5, 541.3)	217.5 (187.5, 326.3)	0.08
Wake after sleep onset min, median (IQR)	0.0 (0.0, 0.0)	0.0 (0.0, 0.0)	1.00
Sleep offset h:mm, median (IQR)	9:00 (7:45, 9:30)	7:00 (6:52, 7:52)	0.04
Wake-up time h:mm, median (IQR)	9:00 (8:00, 9:30)	7:45 (7:22, 8:03)	0.03
Sleep efficiency %, median (IQR)	90.9 (87.9, 94.4)	92.3 (87.5, 93.1)	0.57
Restorative sleep score, mean (SD)	71.5 (12.6)	71.3 (16.2)	0.96
Daytime dysfunction score, mean (SD)	33.4 (21.6)	25.6 (17.7)	0.33
Percentage breakfast intake %, median (IQR)	50.0 (14.3, 71.4)	92.9 (64.3, 92.9)	0.005

*IQR *interquartile range, *MS *midpoint of sleep, *SD *standard deviation, *TST *total sleep time, *VD *vitamin D

### Amount of vitamin D synthesis from UV-B radiation

**Fig. 5:**
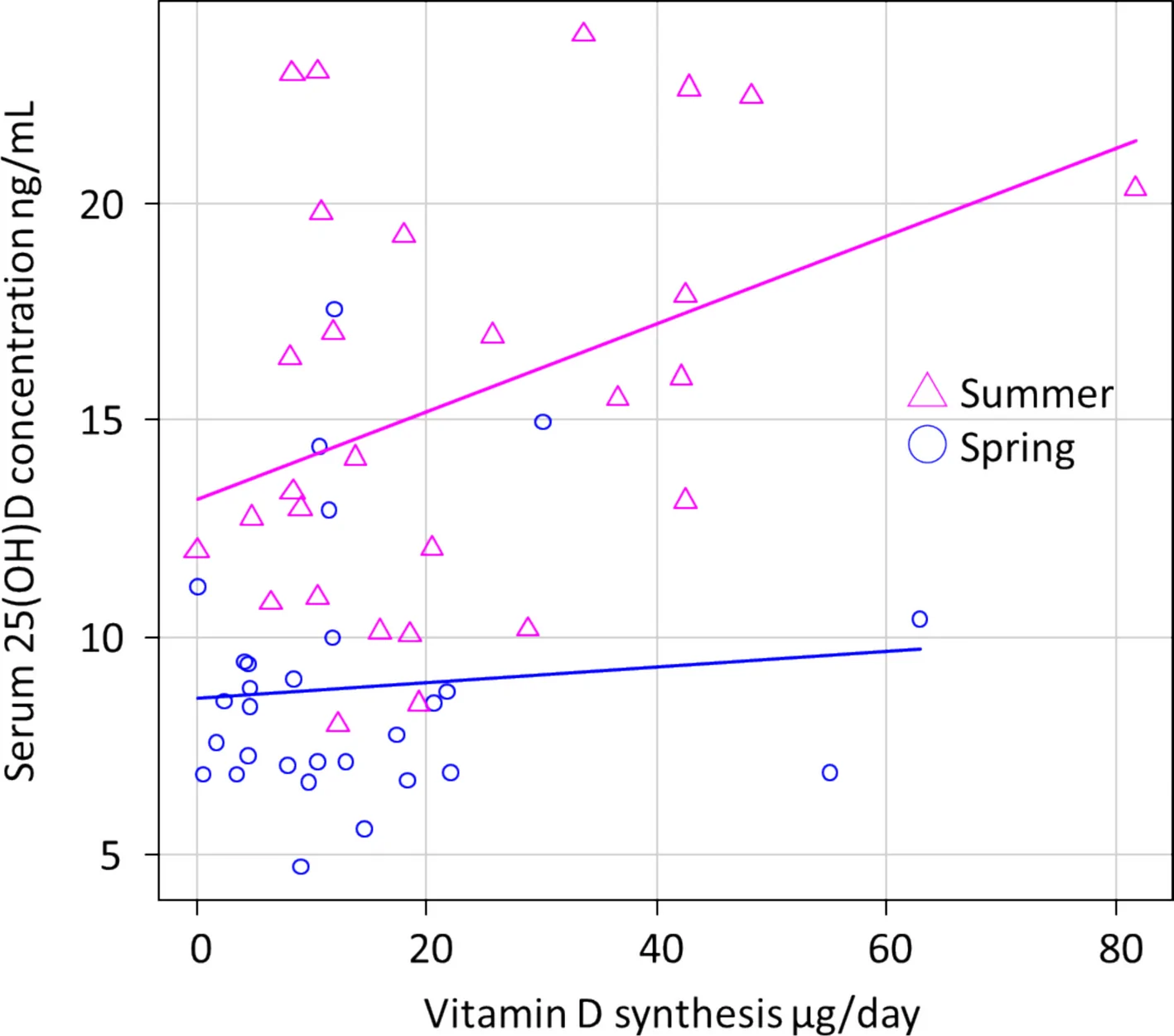
Scatter plot of vitamin D synthesis and serum 25(OH)D concentration. The scatter plot shows the amount of vitamin D synthesized in the skin from UV-B radiation and serum 25(OH)D concentration, separated into spring (○) and summer (△). Spearman's rank correlation coefficients for spring and summer were *ρ* = 0.04 (*P* = 0.85) and *ρ* = 0.27 (*P* = 0.17), respectively. 25(OH)D, 25-hydroxyvitamin D; UV, ultraviolet

**Fig. 6:**
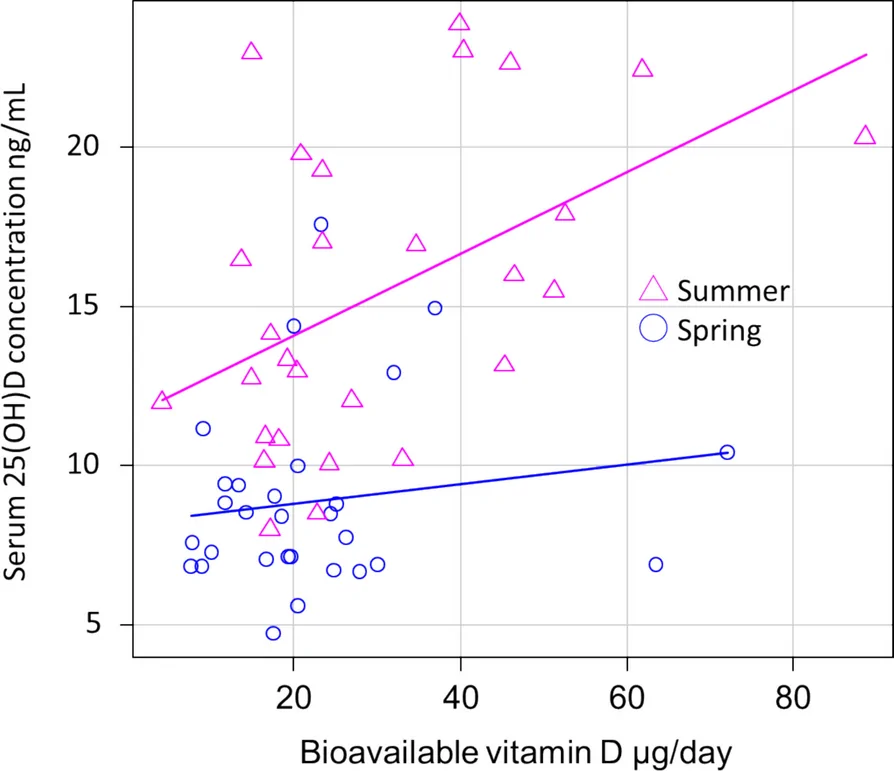
Scatter plot of daily bioavailable vitamin D and serum 25(OH)D concentrations. Daily bioavailable vitamin D was defined as the sum of vitamin D synthesized from daily UV-B exposure and dietary vitamin D intake adjusted for energy intake. The Spearman’s rank correlation coefficients between daily bioavailable vitamin D and serum 25(OH)D concentration were *ρ* = 0.11 (*P* = 0.54) for the spring season and *ρ* = 0.45 (*P* = 0.02) for the summer season. 25(OH)D, 25-hydroxyvitamin D; UV, ultraviolet

The median (IQR) amount of vitamin D synthesis significantly increased from 10.5 (4.6, 17.4) μg/day in spring to 17.0 (10.1, 34.5) μg/day in summer (Wilcoxon rank sum test, *P* = 0.009). The coefficients between the amount of vitamin D synthesis and serum 25(OH)D concentrations were *ρ* = 0.04 (*P* = 0.85) in spring and *ρ* = 0.27 (*P* = 0.17) in summer (Fig. 5). The daily total bioavailable vitamin D was calculated as the sum of vitamin D synthesis and dietary vitamin D intake adjusted for energy intake. The coefficients between the daily bioavailable vitamin D and serum 25(OH)D concentrations were *ρ* = 0.11 (*P* = 0.54) in spring and *ρ* = 0.45 (*P* = 0.02) in summer (Fig. 6).

Similar to vitamin D intake from the diet, we divided participants into two groups based on the median amount of daily vitamin D synthesis and explored factors associated with vitamin D synthesis. Because the amount of vitamin D synthesis is significantly higher in summer than in spring, we divided participants into two groups by the median for both spring and summer to examine each season separately. In spring, the median amount of vitamin D produced was 10.5 μg/day, and participants were divided into two groups accordingly. Compared to the group with vitamin D synthesis < 10.5 μg/day in spring, the group with ≥ 10.5 μg/day had fewer individuals who reported sleep-related symptoms of premenstrual syndrome (see Supplementary Table 4). For summer, the median amount of vitamin D synthesis was 17.0 μg/day, and participants were divided into two groups based on this value. In the group with vitamin D production ≥ 17.0 μg/day, there were more individuals with depressive symptoms, as indicated by a PHQ-9 score ≥ 5 (*P* = 0.03), and the AIS score was also significantly higher (*P* = 0.03) (see Supplementary Table 5).

### Insomnia, depressive symptoms, and vitamin D

**Fig. 7:**
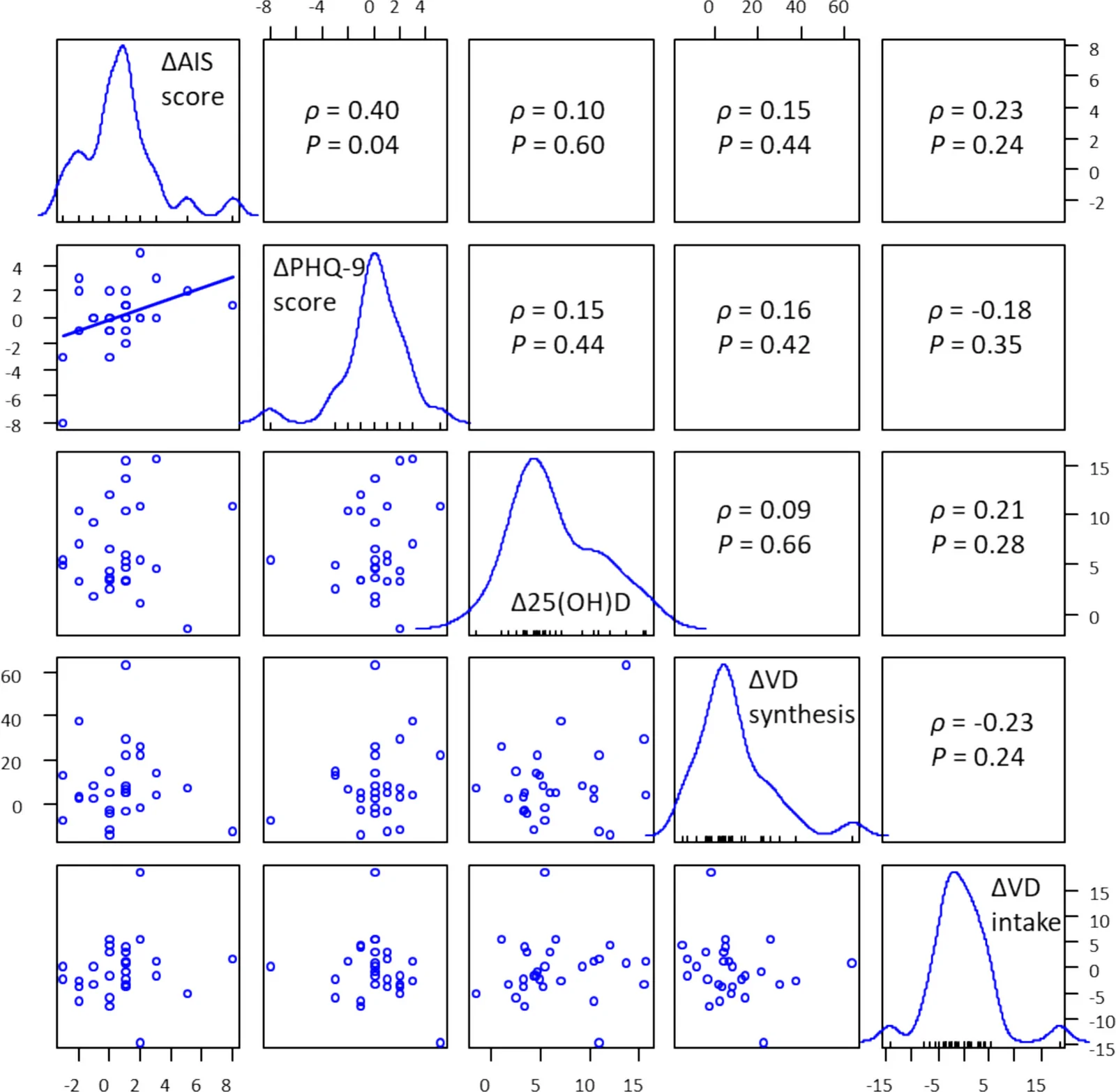
Correlation matrix and scatter plot matrix of changes from spring to summer. The matrices show the correlations and scatter plot matrix of the changes—calculated by subtracting spring values from summer values—in AIS scores, PHQ-9 scores, serum 25(OH)D concentrations, vitamin D synthesis amounts, and vitamin D intake amounts. The correlation matrix is shown in the upper right, the scatter plot matrix in the lower left, and the density plots of each variable are shown on the diagonal. Spearman’s *ρ* and *P* values are indicated in the correlation matrix. For scatter plots where significant correlations were observed, the least squares regression line is also shown. AIS, Athens Insomnia Scale; 25(OH)D, 25-hydroxyvitamin D; PHQ-9, Patient Health Questionnaire-9; VD. vitamin D

In the group with high vitamin D synthesis during the summer, a greater number of individuals exhibited insomnia symptoms with AIS ≥ 6 and depressive symptoms with PHQ-9 ≥ 5, which was confirmed using a scatter plot matrix. The AIS and PHQ-9 scores in summer showed a positive correlation (Spearman’s *ρ* = 0.72, *P* < 0.001); however, neither score was significantly correlated with serum 25(OH)D concentration, dietary vitamin D intake, or vitamin D synthesis (Supplementary Fig. 4). Similarly, when examining the amount of change from spring to summer, the changes in AIS and PHQ-9 scores were also positively correlated (Spearman’s *ρ* = 0.40, *P* = 0.04); however, neither score was significantly correlated with changes in serum 25(OH)D concentration, dietary vitamin D intake, or vitamin D synthesis (Fig. 7). Additionally, no significant correlations were found between changes in serum 25(OH)D concentration and changes in other sleep–wake parameters.

## Discussion

In this study, focusing on young adult Japanese women, it was observed that all participants exhibited vitamin D deficiency during spring. Although this deficiency was ameliorated in the summer, the prevalence of vitamin D deficiency remained notably high. The elevated rate of vitamin D deficiency identified in this study aligns with recent research findings [7–9]. During the summer, there was an observed increase in blood levels, accompanied by a reduction in the number of individuals with severe deficiencies. This highlights the significance of elevated serum vitamin D levels in addressing health issues associated with deficiency. Consequently, we investigated the relationship between alterations in depressive and insomnia symptoms and changes in serum 25(OH)D levels from spring to summer among the participants in this study.

This study explored the potential link between variations in serum 25(OH)D levels, vitamin D consumption, or synthesis, and changes in PHQ-9 or AIS scores. Our analysis did not reveal any significant associations between alterations in vitamin D metrics and scores on these scales. The increase in serum 25(OH)D observed in this study may not have been substantial enough to influence depressive or insomniac symptoms significantly. The impact of vitamin D supplementation on depressive symptoms can be observed within an 8-week period [27]. In this study, although the specific duration during which serum 25(OH)D levels increased was not determined, it may have been essential to sustain elevated blood concentrations for an extended period to effectively mitigate depressive symptoms. Moreover, the administration of vitamin D supplements has been shown to mitigate depressive symptoms in individuals who already present with pronounced depressive symptoms prior to supplementation [27]. In spring, only eight participants had a PHQ-9 score of five or higher, and four participants had an AIS score of six or higher, with even fewer individuals showing relatively high scores. This highlights the importance of focusing on a larger sample of individuals diagnosed with depression or insomnia in future research.

The increase in serum 25(OH)D concentrations from spring to summer was predominantly influenced by vitamin D synthesis from solar UV radiation rather than dietary intake. Nevertheless, only approximately one-quarter of the individuals achieved vitamin D sufficiency, underscoring the need for more proactive interventions. To effectively harness cutaneous vitamin D production via UV exposure, it is imperative to ascertain the levels of UV radiation and exposure duration that do not induce erythema, thereby minimizing the adverse effects of UV radiation. In Japan, data concerning daily erythemal UV radiation and vitamin D-producing UV radiation are accessible online and are derived from actual measurements. This information is utilized to ascertain the appropriate exposure durations [16, 28, 29]. It is desirable to make appropriate use of sunlight without excessively blocking solar UV radiation.

Serum 25(OH)D concentrations are also affected by dietary vitamin D intake. In the Japanese Dietary Reference Intakes (2025), the recommended daily intake of vitamin D has been raised to 9 μg/day [18]. However, in this study, nearly 70% of participants had estimated intake levels (even after adjusting for energy intake) below the recommended amount. As all serum 25(OH)D was D_3_, this suggests that vitamin D_2_ of plant origin was scarcely consumed, highlighting the importance of revising dietary habits to ensure adequate intake of foods rich in vitamin D, such as eggs and fish. The absence of D2 in the blood may also be partly due to its lower bioavailability compared to D_3_ [30, 31].

This study highlights the importance of enhancing lifestyle habits, such as sleep routines, in addition to dietary content, to increase vitamin D intake. Previous studies targeting Japanese female university students and elderly women have reported an association between eveningness, as assessed by the midpoint of sleep, and lower vitamin D intake [32, 33]. Similarly, in the present summer survey, the group with lower vitamin D intake exhibited a delayed midpoint of sleep. Furthermore, this group also lacked habitual breakfast intake. However, these associations were not observed in spring. In summer, a seasonal shift toward earlier midpoints of sleep is observed, which is thought to be related to the light environment and temperature [34]. However, it has also been reported that when a long vacation is included, the midpoint of sleep in summer tends to shift later [35]. In the present study, the participants exhibited a significant delay in the midpoint of sleep during summer by 16.9 min (IQR: − 12.2, 46.3) compared to spring (Wilcoxon signed-rank test, *P* = 0.04). In the summer survey, a more distinct evening-type group emerged, characterized by lower vitamin D intake and a lack of breakfast habits. An evening chronotype has been linked to various physical and mental health issues [36]. Further research involving larger sample sizes is required to ascertain whether an evening chronotype is correlated with breakfast skipping and influences vitamin D deficiency.

This study has some limitations. First, to more accurately monitor skin exposure to UV radiation, the use of portable UV dosimeters is recommended. During actual outdoor activities, individuals are not consistently exposed to direct sunlight; instead, they frequently spend extended periods in shaded areas. Therefore, it is essential to monitor each individual's UV exposure throughout their activities. Second, regarding the assessment of dietary intake, there was a tendency for underreporting in the self-reported survey conducted in this study. Consequently, it was necessary to adjust the intake estimates based on the estimated energy expenditure. Utilizing photographs or other records of each meal is necessary to more accurately estimate the intake. Third, to evaluate the health effects of correcting vitamin D deficiency, it is essential to conduct a study focusing on individuals who are both vitamin D-deficient and have pathological conditions, such as depression or insomnia. Concurrently, it is imperative to determine the optimal increase in serum 25(OH)D levels required. Fourth, it is essential to evaluate sleep–wake parameters through objective methods, such as wearable devices. Given the potential discrepancies between subjective sleep diary parameters and objective assessments, it is advisable to employ both approaches whenever feasible. In this context, the integration of a portable UV dosimeter is anticipated to facilitate more precise monitoring.

## Conclusions

During the spring season, all participants exhibited vitamin D deficiency. Even in the summer, when serum 25(OH)D levels increased mainly due to greater exposure to UV radiation, only 21.4% of the participants achieved sufficient vitamin D levels. At the group level, this increase in serum 25(OH)D levels did not correspond with any noticeable improvement in the symptoms of depression or insomnia. Dietary intake of vitamin D appears to be influenced by breakfast consumption habits, along with delayed sleep phase. Consequently, it is imperative to assess these factors using more objective indicators of lifestyle habits, including sleep–wake patterns, in larger populations. Furthermore, the utilization of UV radiation is crucial for effectively increasing vitamin D levels in the body. However, rather than exhibiting excessive caution towards UV exposure, it is essential to establish a safe framework for UV radiation use that prevents erythema and to verify this approach in a substantial number of cases in future studies.

## Supplementary Information

Supplementary Material 1.

## Data Availability

The datasets used and/or analyzed during the current study are available from the corresponding author upon reasonable request.

## Abbreviations

AIS: Athens Insomnia Scale
CI: Confidence interval
IQR: Interquartile range
LC-MS/MS: Liquid chromatography-tandem mass spectrometry
MST: Mid-sleep time
25(OH)D: 25-Hydroxyvitamin D
PHQ-9: Patient Health Questionnaire-9
PMS: Premenstrual syndrome
PTH: Parathyroid hormone
RLS: Restless legs syndrome
SD: Standard deviation
UV: Ultraviolet
