# Supplementary material for: An exploratory study on the association between vitamin D intake or synthesis and sleep health in young adult women: an observational study
Source: J Physiol Anthropol. 2026 Jul 16;45:22. doi: 10.1186/s40101-026-00436-8 (PMC13656387; doi:10.1186/s40101-026-00436-8)
Supplement: Supplementary file 1 [file 40101_2026_436_MOESM1_ESM.docx]

**Supplementary**

**An exploratory study on the association between vitamin D intake or synthesis and sleep health in young adult women: an observational study**

Rika Yoshizu ^1^, Toru Sasaki ^2^, Hideaki Nakajima ^2^, Hiromi Eto ^3^, Takuya Shuo ^4^, Hideaki Kondo ^5^

1. Department of Reproductive Health, Health Sciences, Graduate School of Biomedical Sciences, Nagasaki University, 1-12-4, Sakamoto, Nagasaki-City, Nagasaki, 852-8523, Japan
2. National Institute for Environmental Studies, 16-2 Onogawa, Tsukuba, Ibaraki 305-8506, Japan
3. Department of Reproductive Health, Institute of Biomedical Sciences, Nagasaki University, 1-7-1 Sakamoto, Nagasaki-city, 852-8520, Japan
4. Faculty of Health and Medical Sciences, Hokuriku University, 1-1 Taiyogaoka, Kanazawa, Ishikawa, 920-1180, Japan
5. Department of General Medicine, Nagasaki University Hospital, 1-7-1, Sakamoto, Nagasaki-City, Nagasaki, 852-8501, Japan

Correspondence: Hideaki Kondo

Department General Medicine, Nagasaki University Hospital

1-7-1 Sakamoto, Nagasaki-city, Nagasaki, 852-8501, Japan

Phone: +/81-95-819-7591, FAX: +81-95-819-7372

E-mail: [kondo_hideaki@nagasaki-u.ac.jp](mailto:kondo_hideaki@nagasaki-u.ac.jp)

**Supplementary Methods**

**Blood collection and test items**

Blood samples were collected in 10-and 5-mL tubes for serum separation. The serum was centrifuged and divided into two tubes. One was frozen at -20 °C and collected by SRL, an external laboratory, on the day of blood collection and transported to the laboratory. The other bottle of serum was stored at -80 °C and transported to Hokuriku University for 25(OH)D measurement using liquid chromatography-tandem mass spectrometry (LC-MS/MS). Total protein, albumin, AST, ALT, Cr, Ca, P, Fe, unsaturated iron-binding capacity (UIBC), ferritin, and intact parathyroid hormone (PTH) levels were measured by the external laboratory. Serum intact PTH levels were measured using an electrochemiluminescence immunoassay. When the serum albumin concentration was < 4 g/dL, the calcium levels were corrected for albumin. The estimated glomerular filtration rate was calculated based on age and Cr values. Transferrin saturation was calculated from serum Fe concentration and UIBC. Serum ferritin levels were measured to assess the influence of iron deficiency on RLS.

**25(OH)D Measurement**

The measurement of 25(OH)D using LC-MS/MS was performed at the Hokuriku University Instrumental Analysis Facility. The 25(OH)D_2_ and 25(OH)D_3_ levels were measured, and 25 (OH) D was used as the sum of the two. Samples were deproteinized in methanol/zinc sulfate as preprocessing before solid-phase extraction was performed using an Oasis PRiME HLB (Waters Corp., MA, US). The LC-MS/MS system used an LCMS-8045 triple-quadrupole mass spectrometer coupled with a Nexera X2 high-performance liquid chromatography system (Shimadzu Corp, Kyoto, Japan); 25(OH)D_2_ and 25(OH)D_3_ were separated on a reverse-phase column (Shim-pack Velox SP-C18, Shimadzu Corp, Kyoto, Japan) and underwent electrospray ionization before the assay was performed using multiple reaction monitoring. The 25(OH)D_2_ and 25(OH)D_3_ calibrators and deuterated internal standards used were CertiMass Reference Standards (IsoSciences LLC, Ambler, PA, US). Quality control for 25(OH)D_2_ and 25(OH)D_3_ serum level measurements were performed using the ClinCal Serum Calibrator Set (RECIPE, München, Germany).

**Supplementary information on the dietary intake survey**

In the dietary intake survey, the following data processing was conducted. When the absolute difference between the reported energy intake and the energy intake estimated from body weight exceeds 30% of the energy expenditure estimated from body weight, it is empirically considered advisable to refrain from using this data for nutrition and dietary guidance. Therefore, participants with an estimated reporting error of 30% or more were classified into the over-reporting/under-reporting group, whereas those with less than 30% were classified into the appropriate reporting group. In addition, the proportions of carbohydrates, protein, fat, and alcohol—nutrients that provide energy—relative to total energy intake were calculated.

**Questionnaire on premenstrual syndrome**

A questionnaire assessing premenstrual syndrome evaluated symptoms related to physical, psychological, and sleep-wake domains. Physical symptoms encompassed headache, abdominal bloating, swelling of the extremities, and breast pain. Psychological symptoms included depression, anger outbursts, irritability, anxiety, confusion, and social withdrawal. Regarding sleep and wakefulness, the questionnaire assessed difficulty in falling asleep, nocturnal awakenings, leg discomfort, and daytime sleepiness. In the free description section, numerous participants reported abdominal pain, and those reporting this symptom were categorized under physical symptoms. Participants who experienced symptoms within the five days preceding menstruation, persisting for more than three months, with symptoms resolving within four days of menstrual onset and not reappearing by the thirteenth day post-menstruation, were classified as having premenstrual syndrome. All other participants were classified as not having premenstrual syndrome.

Supplementary Figure 1. Scatter plot of energy and vitamin D intake based on the dietary survey. Spearman’s rank correlation coefficient between energy and vitamin D intake was *ρ* = 0.41 (*P* = 0.002).

Supplementary Figure 2. Scatter plot of serum 25(OH)D concentration and serum intact PTH concentration. Spring is indicated by (○) and summer by (△). No significant correlation was observed between serum 25(OH)D concentration and serum intact PTH concentration (Spearman’s rank correlation coefficient *ρ* = -0.25, *P* = 0.06). 25(OH)D, 25-hydroxyvitamin D; PTH, parathyroid hormone.

Supplementary Table 1. Comparison between two groups with summer serum 25(OH)D concentrations < 15 ng/mL and ≥ 15 ng/mL

|  | 25(OH)D < 15 ng/mL | 25(OH)D ≥ 15 ng/mL | *P* value |
| --- | --- | --- | --- |
| n | 14 | 14 |  |
| age, median (IQR) | 23.5 (22.3, 26.5) | 23.0 (22.0, 24.0) | 0.35 |
| BMI kg/m^2^, median (IQR) | 21.3 (20.4, 21.6) | 19.7 (18.7, 22.4) | 0.23 |
| ≥ 25 kg/m^2^, n (%) | 2 (14.3) | 1 (7.1) | 1.00 |
| Living alone, n (%) | 10 (71.4) | 9 (64.3) | 1.00 |
| Non-smoking, n (%) | 3 (21.4) | 5 (35.7) | 0.68 |
| Regular exercise, n (%) | 14 (100.0) | 14 (100.0) | 1.00 |
| Diet |  |  |  |
| Energy intake kcal, median (IQR) | 1433.4 (1204.5, 1629.2) | 1436.1 (1371.5, 1605.1) | 0.71 |
| Carbohydrate %, median (IQR) | 50.2 (47.8, 57.4) | 50.2 (48.8, 54.2) | 0.89 |
| Protein %, median (IQR) | 12.8 (11.7, 14.6) | 13.9 (12.1, 14.6) | 0.82 |
| Fat %, median (IQR) | 30.0 (25.3, 32.5) | 29.6 (26.9, 33.3) | 0.75 |
| Alcohol %, median (IQR) | 0.3 (0.0, 7.3) | 3.1 (0.1, 6.1) | 0.64 |
| Habitual drinking, n (%) | 5 (35.7) | 6 (42.9) | 1.00 |
| VD intake μg/day, median (IQR) | 3.5 (2.4, 5.9) | 5.9 (4.8, 8.4) | 0.05 |
| VD intake μg/day/1,000 kcal, median (IQR) | 2.6 (1.7, 4.7) | 3.8 (3.2, 5.1) | 0.10 |
| Estimated energy intake kcal, median (IQR) | 2042.5 (1919.0, 2090.0) | 1900.0 (1795.5, 2141.3) | 0.43 |
| Reporting error rate %, median (IQR) | -22.8 (-45.0, -15.8) | -23.3 (-40.0, -10.5) | 0.61 |
| Over / Under reporting, n (%) | 9 (64.3) | 9 (64.3) | 1.00 |
| Corrected VD intake μg/day, median (IQR) | 5.2 (3.6, 9.1) | 8.0 (5.6, 11.2) | 0.08 |
| Corrected VD intake ≥ 9 μg/day, n (%) | 4 (28.6) | 7 (50.0) | 0.44 |
| Average VD synthesis μg/day, median (IQR) | 13.1 (8.5, 19.1) | 29.8 (11.1, 42.5) | 0.06 |
| Average VD synthesis ≥ 10 μg/day, n (%) | 5 (35.7) | 9 (64.3) | 0.26 |
| Serum concentration |  |  |  |
| 25(OH)D3 ng/mL, median (IQR) | 12.6 (11.0, 13.9) | 16.7 (15.2, 21.5) | < 0.001 |
| Intact-PTH pg/mL, median (IQR) | 33.0 (28.5, 40.5) | 31.0 (26.3, 47.8) | 0.95 |
| Corrected Ca mg/dL, median (IQR) | 9.1 (8.9, 9.4) | 9.4 (9.2, 9.5) | 0.03 |
| P mg/dL, median (IQR) | 3.6 (3.2, 3.7) | 3.6 (3.3, 3.9) | 0.85 |
| PMS, n (%) | 9 (64.3) | 10 (71.4) | 1.00 |
| Physical symptoms, n (%) | 10 (71.4) | 11 (78.6) | 1.00 |
| Mental symptoms, n (%) | 5 (35.7) | 9 (64.3) | 0.26 |
| Sleep-related symptoms, n (%) | 8 (57.1) | 7 (50.0) | 1.00 |
| Use of oral contraceptives, n (%) | 1 (7.1) | 4 (28.6) | 0.33 |
| PHQ-9 score, median (IQR) | 1.5 (1.0, 3.0) | 3.0 (1.0, 6.0) | 0.15 |
| ≥ 5, n (%) | 2 (14.3) | 6 (42.9) | 0.21 |
| AIS score, median (IQR) | 3.0 (2.0, 4.0) | 3.5 (2.0, 5.0) | 0.56 |
| ≥ 6, n (%) | 3 (21.4) | 2 (14.3) | 1.00 |
| Difficulty of initiating sleep, n (%) | 7 (50.0) | 7 (50.0) | 1.00 |
| Difficulty of maintaining sleep, n (%) | 0 (0.0) | 3 (21.4) | 0.22 |
| Early wakening, n %) | 0 (0.0) | 4 (28.6) | 0.10 |
| Daytime sleepiness, n (%) | 8 (57.1) | 12 (85.7) | 0.21 |
| RLS, n (%) | 4 (28.6) | 3 (21.4) | 1.00 |
| Sleep diary |  |  |  |
| Total sleep time (TST) hr, median (IQR) | 6.56 (6.00, 7.00) | 6.00 (5.56, 6.75) | 0.26 |
| < 6 hr, n (%) | 1 (7.1) | 2 (14.3) | 0.41 |
| 6–7 hr. n (%) | 5 (35.7) | 8 (57.1) |  |
| ≥ 7 hr, n (%) | 8 (57.1) | 4 (28.6) |  |
| Range of TST hr, median (IQR) | 5.25 (3.63, 6.75) | 3.00 (2.50, 4.00) | 0.03 |
| Irregularity of TST hr/week, median (IQR) | 7.88 (5.31, 11.12) | 4.79 (3.81, 6.34) | 0.03 |
| Bedtime h:mm, median (IQR) | 1:37 (1:00, 2:05) | 0:37 (23:48, 1:45) | 0.07 |
| Sleep latency min, median (IQR) | 26.3 (3.8, 30.0) | 30.0 (15.0, 30.0) | 0.73 |
| Sleep onset h:mm, median (IQR) | 2:00 (1:33, 2:41) | 1:00 (0:11, 2:11) | 0.07 |
| MS h:mm, median (IQR) | 5:33 (4:40, 5:58) | 4:00 (3:30, 5:07) | 0.005 |
| Irregularity of MS min/week, median (IQR) | 320.6 (266.3, 466.9) | 243.8 (198.8, 334.7) | 0.20 |
| Wake after sleep onset min, median (IQR) | 0.00 (0.00, 0.00) | 0.00 (0.00, 0.00) | 1 |
| Sleep offset h:mm, median (IQR) | 9:00 (8:15, 9:22) | 7:05 (6:33, 7:30) | 0.008 |
| Wake-up time h:mm, median (IQR) | 9:00 (8:20, 9:30) | 7:37 (7:00, 8:00) | 0.008 |
| Sleep efficiency %, median (IQR) | 92.0 (88.3, 93.8) | 89.8 (86.6, 92.7) | 0.31 |
| Restorative sleep score, mean (SD) | 70.0 (13.0) | 72.9 (15.0) | 0.60 |
| Daytime dysfunction score, mean (SD) | 34.5 (23.7) | 26.2 (15.8) | 0.28 |
| Percentage breakfast intake %, median (IQR) | 60.7 (17.9, 91.1) | 67.9 (51.8, 78.6) | 0.63 |

AIS, Athens Insomnia Scale; BMI, body mass index; IQR, interquartile range; 25(OH)D, 25-hydroxyvitamin D; MS, midpoint of sleep; PHQ-9, Patient Health Questionnaire-9; PMS, premenstrual syndrome; PTH, parathyroid hormone; RLS, restless legs syndrome; SD, standard deviation; TST, total sleep time; VD, vitamin D.

a. Vitamin D intake	b. Vitamin D synthesis

Supplementary Figure 3. Scatter plots illustrating the relationship between changes in serum 25(OH)D concentrations and alterations in vitamin D intake, as well as changes in vitamin D synthesis (b). a: The Spearman’s correlation coefficient (ρ) between changes in serum 25(OH)D concentration and changes in dietary vitamin D intake, as determined by dietary surveys, was 0.09 (*P* = 0.66). b: Similarly, the correlation between changes in vitamin D synthesis due to UV-B exposure to the skin was 0.21 (*P* = 0.28). In both instances, no significant correlations were observed. 25(OH)D, 25-hydroxyvitamin D; UV, ultraviolet.

Supplementary Table 2. Comparison between two groups with spring vitamin D intake < 7.5 μg/day and ≥ 7.5 μg/day

|  | VD intake < 7.5 μg/day | VD intake ≥ 7.5 μg/day | *P* value |
| --- | --- | --- | --- |
| n | 11 | 18 |  |
| Age, median (IQR) | 23.0 (22.5, 24.0) | 22.5 (21.0, 23.8) | 0.40 |
| BMI kg/m^2^, median (IQR) | 20.6 (19.5, 22.3) | 20.8 (18.9, 21.7) | 0.70 |
| ≥ 25 kg/m^2^, n (%) | 1 (9.1) | 1 (5.6) | 1.00 |
| Living alone, n (%) | 8 (72.7) | 13 (72.2) | 1.00 |
| Non-smoking, n (%) | 11 (100.0) | 18 (100.0) | 1.00 |
| Regular exercise, n (%) | 5 (45.5) | 9 (50.0) | 1.00 |
| Diet |  |  |  |
| Energy intake kcal, median (IQR) | 1541.9 (1242.6, 1767.6) | 1379.4 (1142.2, 1526.6) | 0.45 |
| Carbohydrate %, median (IQR) | 52.4 (46.1, 55.2) | 47.7 (43.6, 51.8) | 0.32 |
| Protein %, median (IQR) | 12.3 (11.8, 14.4) | 15.0 (13.0, 16.5) | 0.04 |
| Fat %, median (IQR) | 29.8 (24.3, 34.7) | 31.6 (23.4, 34.1) | 0.79 |
| Alcohol %, median (IQR) | 6.1 (0.0, 8.2) | 0.5 (0.0, 4.1) | 0.55 |
| Habitual drinking, n (%) | 6 (54.5) | 6 (33.3) | 0.44 |
| VD intake μg/day, median (IQR) | 4.1 (3.0, 5.3) | 5.9 (5.1, 9.0) | 0.01 |
| VD intake μg/day/1,000 kcal, median (IQR) | 3.0 (2.5, 3.3) | 4.4 (4.0, 5.7) | < 0.001 |
| Estimated energy intake kcal, median (IQR) | 1900.0 (1824.0, 2147.0) | 2014.0 (1852.5, 2085.3) | 1.00 |
| Reporting error rate %, median (IQR) | -20.2 (-39.2, -9.2) | -30.1 (-40.1, -24.1) | 0.30 |
| Over / Under reporting, n (%) | 7 (63.6) | 8 (44.4) | 0.45 |
| Corrected VD intake μg/day, median (IQR) | 6.1 (5.4, 6.7) | 9.0 (8.6, 10.8) | < 0.001 |
| Corrected VD intake ≥ 9 μg/day, n (%) | 0 (0.0) | 9 (50.0) | 0.005 |
| Average VD synthesis μg/day, median (IQR) | 13.0 (4.0, 19.6) | 10.1 (5.5, 12.0) | 0.89 |
| Average VD synthesis ≥ 10 μg/day, n (%) | 6 (54.5) | 9 (50.0) | 1.00 |
| Serum concentration |  |  | 0 |
| 25(OH)D3 ng/mL, median (IQR) | 7.3 (6.9, 8.6) | 8.8 (7.1, 10.3) | 0.15 |
| Intact-PTH pg/mL, median (IQR) | 38.0 (37.0, 46.0) | 43.5 (40.3, 56.3) | 0.44 |
| Corrected Ca mg/dL, median (IQR) | 9.1 (9.0, 9.4) | 9.3 (9.1, 9.4) | 0.46 |
| P mg/dL, median (IQR) | 3.7 (3.5, 3.9) | 3.8 (3.6, 4.2) | 0.14 |
| PMS, n (%) | 7 (63.6) | 13 (72.2) | 0.69 |
| Physical symptoms, n (%) | 8 (72.7) | 16 (88.9) | 0.34 |
| Mental symptoms, n (%) | 3 (27.3) | 12 (66.7) | 0.06 |
| Sleep-related symptoms, n (%) | 6 (54.5) | 10 (55.6) | 1.00 |
| Use of oral contraceptives, n (%) | 2 (18.2) | 2 (11.1) | 0.62 |
| PHQ-9 score, median (IQR) | 3.0 (1.0, 5.0) | 1.0 (1.0, 4.8) | 0.48 |
| ≥ 5, n (%) | 3 (27.3) | 5 (27.8) | 1.00 |
| AIS score, median (IQR) | 2.0 (2.0, 5.0) | 2.0 (1.0, 3.0) | 0.40 |
| ≥ 6, n (%) | 2 (18.2) | 2 (11.1) | 0.62 |
| Difficulty of initiating sleep, n (%) | 4 (36.4) | 9 (50.0) | 0.70 |
| Difficulty of maintaining sleep, n (%) | 1 (9.1) | 1 (5.6) | 1.00 |
| Early wakening, n %) | 1 (9.1) | 2 (11.1) | 1.00 |
| Daytime sleepiness, n (%) | 9 (81.8) | 10 (55.6) | 0.23 |
| RLS, n (%) | 2 (18.2) | 4 (22.2) | 1.00 |
| Sleep diary |  |  |  |
| TST hr, median (IQR) | 6.00 (5.85, 6.75) | 7.00 (6.50, 7.44) | 0.17 |
| < 6 hr, n (%) | 3 (27.3) | 1 (5.6) | 0.15 |
| 6–7 hr. n (%) | 4 (36.4) | 12 (66.7) |  |
| ≥ 7 hr, n (%) | 4 (36.4) | 5 (27.8) |  |
| Range of TST hr, median (IQR) | 6.00 (3.75, 7.25) | 4.25 (3.00, 5.98) | 0.27 |
| Irregularity of TST hr/week, median (IQR) | 6.83 (5.25, 9.85) | 5.71 (3.87, 8.56) | 0.35 |
| Bedtime h:mm, median (IQR) | 0:45 (0:15, 1:30) | 1:00 (23:33, 1:00) | 0.41 |
| Sleep latency min, median (IQR) | 15.0 (0.0, 30.0) | 27.5 (13.1, 52.5) | 0.17 |
| Sleep onset h:mm, median (IQR) | 1:30 (0:37, 2:30) | 2:00 (0:45, 2:30) | 0.89 |
| MS h:mm, median (IQR) | 4:15 (4:11, 5:07) | 4:37 (3:39, 5:26) | 0.89 |
| Irregularity of MS min/week, median (IQR) | 360.0 (238.1, 453.8) | 247.5 (172.5, 375.0) | 0.25 |
| Wake after sleep onset min, median (IQR) | 0.0 (0.0, 0.0) | 0.0 (0.0, 0.0) | 0.07 |
| Sleep offset h:mm, median (IQR) | 7:30 (7:05, 8:07) | 8:07 (7:00, 9:18) | 0.54 |
| Wake-up time h:mm, median (IQR) | 8:00 (7:26, 8:37) | 8:07 (7:30, 9:52) | 0.62 |
| Sleep efficiency %, median (IQR) | 92.6 (89.7, 94.0) | 88.0 (82.3, 91.1) | 0.10 |
| Restorative sleep score, mean (SD) | 74.2 (11.6) | 65.9 (10.1) | 0.05 |
| Daytime dysfunction score, mean (SD) | 33.3 (16.6) | 24.2 (21.3) | 0.24 |
| Percentage breakfast intake %, median (IQR) | 57.1 (17.9, 71.4) | 60.7 (37.5, 85.7) | 0.46 |

AIS, Athens Insomnia Scale; BMI, body mass index; IQR, interquartile range; 25(OH)D, 25-hydroxyvitamin D; MS, midpoint of sleep; PHQ-9, Patient Health Questionnaire-9; PMS, premenstrual syndrome; PTH, parathyroid hormone; RLS, restless legs syndrome; SD, standard deviation; TST, total sleep time; VD, vitamin D.

Supplementary Table 3. Comparison between two groups with summer vitamin D intake of < 7.5 μg/day and ≥ 7.5 μg/day

|  | VD intake < 7.5 μg/day | VD intake ≥ 7.5 μg/day | *P* value |
| --- | --- | --- | --- |
| n | 17 | 11 |  |
| age, median (IQR) | 23.0 (22.0, 25.0) | 24.0 (23.0, 24.0) | 0.42 |
| BMI kg/m^2^, median (IQR) | 21.2 (20.3, 21.6) | 19.9 (18.5, 22.3) | 0.25 |
| ≥ 25 kg/m^2^, n (%) | 2 (11.8) | 1 (9.1) | 1.00 |
| Living alone, n (%) | 14 (82.4) | 5 (45.5) | 0.10 |
| Non-smoking, n (%) | 17 (100.0) | 11 (100.0) | 1.00 |
| Regular exercise, n (%) | 5 (29.4) | 7 (63.6) | 0.12 |
| Diet |  |  |  |
| Energy intake kcal, median (IQR) | 1449.9 (1375.6, 1759.6) | 1431.6 (1283.2, 1504.4) | 0.38 |
| Carbohydrate %, median (IQR) | 54.4 (49.6, 58.0) | 48.7 (48.3, 49.4) | 0.01 |
| Protein %, median (IQR) | 12.1 (11.2, 12.8) | 14.7 (14.4, 16.4) | < 0.001 |
| Fat %, median (IQR) | 27.3 (22.2, 31.8) | 31.9 (29.0, 33.3) | 0.07 |
| Alcohol %, median (IQR) | 3.5 (0.1, 7.7) | 0.2 (0.0, 3.7) | 0.23 |
| Habitual drinking, n (%) | 8 (47.1) | 3 (27.3) | 0.44 |
| VD intake μg/day, median (IQR) | 3.2 (2.4, 4.7) | 8.5 (6.8, 9.4) | < 0.001 |
| VD intake μg/day/1,000 kcal, median (IQR) | 2.3 (1.7, 3.1) | 5.5 (5.1, 6.9) | < 0.001 |
| Estimated energy intake kcal, median (IQR) | 2033.0 (1900.0, 2090.0) | 1900.0 (1786.0, 2154.6) | 0.41 |
| Reporting error rate %, median (IQR) | -22.8 (-40.6, -13.5) | -23.4 (-39.8, -18.1) | 0.56 |
| Over / Under reporting, n (%) | 11 (64.7) | 7 (63.6) | 1 |
| Corrected VD intake μg/day, median (IQR) | 4.7 (3.5, 6.0) | 11.4 (10.0, 12.7) | < 0.001 |
| Corrected VD intake ≥ 9 μg/day, n (%) | 0 (0.0) | 10 (90.9) | < 0.001 |
| Average VD synthesis μg/day, median (IQR) | 18.6 (12.3, 33.8) | 10.9 (8.7, 31.2) | 0.36 |
| Average VD synthesis ≥ 10 μg/day, n (%) | 14 (82.4) | 7 (63.6) | 0.38 |
| Serum concentration |  |  |  |
| 25(OH)D3 ng/mL, median (IQR) | 13.2 (10.2, 19.3) | 16.9 (13.2, 18.8) | 0.23 |
| Intact-PTH pg/mL, median (IQR) | 32.0 (29.0, 42.0) | 30.0 (24.5, 41.0) | 0.30 |
| Corrected Ca mg/dL, median (IQR) | 9.2 (9.0, 9.4) | 9.4 (9.2, 9.5) | 0.41 |
| P mg/dL, median (IQR) | 3.6 (3.3, 3.7) | 3.4 (3.3, 3.8) | 0.49 |
| PMS, n (%) | 11 (64.7) | 8 (72.7) | 1.00 |
| Physical symptoms, n (%) | 12 (70.6) | 9 (81.8) | 0.67 |
| Mental symptoms, n (%) | 9 (52.9) | 5 (45.5) | 1.00 |
| Sleep-related symptoms, n (%) | 9 (52.9) | 6 (54.5) | 1.00 |
| Use of oral contraceptives, n (%) | 3 (17.6) | 2 (18.2) | 1.00 |
| PHQ-9 score, median (IQR) | 3.0 (1.0, 6.0) | 1.0 (1.0, 3.5) | 0.39 |
| ≥ 5, n (%) | 6 (35.3) | 2 (18.2) | 0.42 |
| AIS score, median (IQR) | 3.0 (2.0, 5.0) | 3.0 (1.5, 4.5) | 0.83 |
| ≥ 6, n (%) | 3 (17.6) | 2 (18.2) | 1.00 |
| Difficulty of initiating sleep, n (%) | 10 (58.8) | 4 (36.4) | 0.44 |
| Difficulty of maintaining sleep, n (%) | 1 (5.9) | 2 (18.2) | 0.54 |
| Early wakening, n %) | 1 (5.9) | 3 (27.3) | 0.27 |
| Daytime sleepiness, n (%) | 12 (70.6) | 8 (72.7) | 1.00 |
| RLS, n (%) | 5 (29.4) | 2 (18.2) | 0.67 |

AIS, Athens Insomnia Scale; BMI, body mass index; IQR, interquartile range; 25(OH)D, 25-hydroxyvitamin D; PHQ-9, Patient Health Questionnaire-9; PMS, premenstrual syndrome; PTH, parathyroid hormone; RLS, restless legs syndrome; SD, standard deviation; VD, vitamin D.

Supplementary Table 4. Comparison between the two groups with spring vitamin D synthesis of < 10.5 μg/day and ≥ 10.5 μg/day

|  | VD synthesis < 10.5 μg/day | VD synthesis ≥ 10.5 μg/day | *P* value |
| --- | --- | --- | --- |
| n | 14 | 15 |  |
| age, median (IQR) | 23.0 (21.0, 24.0) | 23.0 (21.5, 23.0) | 0.64 |
| BMI kg/m^2^, median (IQR) | 21.0 (18.9, 21.5) | 20.6 (19.5, 22.3) | 0.76 |
| ≥ 25 kg/m^2^, n (%) | 0 (0.0) | 2 (13.3) | 0.48 |
| Living alone, n (%) | 11 (78.6) | 10 (66.7) | 0.68 |
| Non-smoking, n (%) | 14 (100.0) | 15 (100.0) | 1.00 |
| Regular exercise, n (%) | 4 (28.6) | 10 (66.7) | 0.07 |
| Diet |  |  |  |
| Energy intake kcal, median (IQR) | 1424.7 (1098.2, 1677.5) | 1387.3 (1251.1, 1572.6) | 1.00 |
| Carbohydrate %, median (IQR) | 52.0 (46.1, 60.4) | 46.4 (44.1, 50.7) | 0.15 |
| Protein %, median (IQR) | 14.7 (12.4, 16.1) | 13.3 (12.7, 15.8) | 0.83 |
| Fat %, median (IQR) | 28.5 (23.4, 33.9) | 31.9 (24.3, 36.2) | 0.46 |
| Alcohol %, median (IQR) | 0.0 (0.0, 1.5) | 6.1 (0.3, 9.6) | 0.03 |
| Habitual drinking, n (%) | 3 (21.4) | 9 (60.0) | 0.06 |
| VD intake μg/day, median (IQR) | 5.6 (4.1, 8.2) | 5.2 (4.0, 6.4) | 0.73 |
| VD intake μg/day/1,000 kcal, median (IQR) | 4.1 (3.3, 5.0) | 3.9 (3.2, 4.6) | 0.49 |
| Estimated energy intake kcal, median (IQR) | 2004.5 (1843.0, 2090.0) | 2014.0 (1824.0, 2147.0) | 0.97 |
| Reporting error rate %, median (IQR) | -29.2 (-43.1 -11.2) | -26.7 (-39.2, -20.6) | 0.90 |
| Over / Under reporting, n (%) | 6 (42.9) | 9 (60.0) | 0.47 |
| Corrected VD intake μg/day, median (IQR) | 8.6 (6.9, 9.2) | 8.4 (6.4, 9.1) | 0.63 |
| Corrected VD intake ≥ 9 μg/day, n (%) | 5 (35.7) | 4 (26.7) | 0.70 |
| Average VD synthesis μg/day, median (IQR) | 4.5 (2.7, 7.1) | 17.4 (11.9, 21.9) | < 0.001 |
| Average VD synthesis ≥ 10 μg/day, n (%) | 0 (0.0) | 15 (100.0) | < 0.001 |
| Serum concentration |  |  | 0 |
| 25(OH)D3 ng/mL, median (IQR) | 8.0 (6.9, 9.0) | 8.5 (7.0, 11.7) | 0.34 |
| Intact-PTH pg/mL, median (IQR) | 42.0 (35.8, 56.3) | 43.0 (38.0, 49.0) | 1.00 |
| Corrected Ca mg/dL, median (IQR) | 9.4 (9.2, 9.5) | 9.1 (8.9, 9.3) | 0.03 |
| P mg/dL, median (IQR) | 3.8 (3.7, 4.0) | 3.7 (3.5, 3.9) | 0.20 |
| PMS, n (%) | 10 (71.4) | 10 (66.7) | 1.00 |
| Physical symptoms, n (%) | 13 (92.9) | 11 (73.3) | 0.33 |
| Mental symptoms, n (%) | 8 (57.1) | 7 (46.7) | 0.72 |
| Sleep-related symptoms, n (%) | 11 (78.6) | 5 (33.3) | 0.03 |
| Use of oral contraceptives, n (%) | 0 (0.0) | 4 (26.7) | 0.10 |
| PHQ-9 score, median (IQR) | 3.0 (0.0, 4.8) | 1.0 (1.0, 4.5) | 0.58 |
| ≥ 5, n (%) | 4 (28.6) | 4 (26.7) | 1.00 |
| AIS score, median (IQR) | 2.5 (1.3, 4.8) | 2.0 (1.0, 3.0) | 0.63 |
| ≥ 6, n (%) | 3 (21.4) | 1 (6.7) | 0.33 |
| Difficulty of initiating sleep, n (%) | 8 (57.1) | 5 (33.3) | 0.27 |
| Difficulty of maintaining sleep, n (%) | 1 (7.1) | 1 (6.7) | 1.00 |
| Early wakening, n %) | 1 (7.1) | 2 (13.3) | 1.00 |
| Daytime sleepiness, n (%) | 9 (64.3) | 10 (66.7) | 1.00 |
| RLS, n (%) | 2 (14.3) | 4 (26.7) | 0.65 |
| Sleep diary |  |  | 0 |
| Total sleep time (TST) hr, median (IQR) | 6.75 (6.06, 7.44) | 6.50 (5.81, 7.17) | 0.37 |
| < 6 hr, n (%) | 1 (7.1) | 3 (20.0) | 0.68 |
| 6–7 hr. n (%) | 9 (64.3) | 7 (46.7) | 0 |
| ≥ 7 hr, n (%) | 4 (28.6) | 5 (33.3) | 0 |
| Range of TST hr, median (IQR) | 4.25 (3.12, 5.37) | 6.50 (3.25, 7.25) | 0.14 |
| Irregularity of TST hr/week, median (IQR) | 6.00 (4.56, 8.50) | 6.50 (4.37, 11.42) | 0.54 |
| Bedtime h:mm, median (IQR) | 1:00 (0:18, 1:00) | 0:30 (23:45, 1:15) | 0.37 |
| Sleep latency min, median (IQR) | 30.0 (13.1, 30.0) | 15.0 (0.0, 30.0) | 0.22 |
| Sleep onset h:mm, median (IQR) | 1:02 (0:45, 2:11) | 1:00 (0:15, 1:52) | 0.41 |
| MS h:mm, median (IQR) | 4:46 (4:09, 5:26) | 4:15 (3:30, 5:03) | 0.23 |
| Irregularity of MS min/week, median (IQR) | 278.75 (220.31, 393.75) | 247.50 (187.50, 398.13) | 0.98 |
| Wake after sleep onset min, median (IQR) | 0.0 (0.0, 0.0) | 0.0 (0.0, 0.0) | 0.92 |
| Sleep offset h:mm, median (IQR) | 8:30 (7:18, 9:41) | 7:30 (6:52, 8:07) | 0.11 |
| Wake-up time h:mm, median (IQR) | 8:37 (7:33, 10:00) | 7:45 (7:26, 8:30) | 0.10 |
| Sleep efficiency %, median (IQR) | 90.4 (86.7, 92.6) | 87.9 (81.0, 93.0) | 0.86 |
| Restorative sleep score, mean (SD) | 69.4 (10.7) | 68.7 (12.1) | 0.86 |
| Daytime dysfunction score, mean (SD) | 30.0 (21.1 | 25.4 (19.0) | 0.54 |
| Percentage breakfast intake %, median (IQR) | 57.1 (10.7, 78.6) | 64.3 (39.3, 89.29) | 0.36 |

AIS, Athens Insomnia Scale; BMI, body mass index; IQR, interquartile range; 25(OH)D, 25-hydroxyvitamin D; MS, midpoint of sleep; PHQ-9, Patient Health Questionnaire-9; PMS, premenstrual syndrome; PTH, parathyroid hormone; RLS, restless legs syndrome; SD, standard deviation; TST, total sleep time; VD, vitamin D.

Supplementary Table 5. Comparison between the two groups with summer vitamin D synthesis of < 17.0 μg/day and ≥ 17.0 μg/day

|  | VD synthesis < 17.0 μg/day | VD synthesis ≥ 17.0 μg/day | *P* value |
| --- | --- | --- | --- |
| n | 14 | 14 |  |
| age, median (IQR) | 24.0 (23.0, 26.5) | 23.0 (22.0, 23.8) | 0.06 |
| BMI kg/m^2^, median (IQR) | 20.5 (18.7, 21.6) | 20.9 (19.7, 22.8) | 0.40 |
| ≥ 25 kg/m^2^, n (%) | 1 (7.1) | 2 (14.3) | 1.00 |
| Living alone, n (%) | 9 (64.3) | 10 (71.4) | 1.00 |
| Non-smoking, n (%) | 14 (100.0) | 14 (100.0) | 1.00 |
| Regular exercise, n (%) | 6 (42.9) | 6 (42.9) | 1.00 |
| Diet |  |  |  |
| Energy intake kcal, median (IQR) | 1376.8 (1246.3, 1522.5) | 1543.3 (1423.9, 1888.3) | 0.04 |
| Carbohydrate %, median (IQR) | 49.5 (48.3, 54.9) | 50.9 (48.8, 56.9) | 0.71 |
| Protein %, median (IQR) | 14.2 (12.2, 15.8) | 12.6 (11.1, 14.3) | 0.07 |
| Fat %, median (IQR) | 29.7 (25.9, 31.6) | 30.5 (23.5, 33.3) | 0.71 |
| Alcohol %, median (IQR) | 1.1 (0.0, 6.6) | 3.7 (0.1, 6.3) | 0.71 |
| Habitual drinking, n (%) | 4 (28.6) | 7 (50.0) | 0.44 |
| VD intake μg/day, median (IQR) | 5.5 (3.2, 7.6) | 5.0 (2.8, 7.4) | 0.71 |
| VD intake μg/day/1,000 kcal, median (IQR) | 4.2 (2.4, 5.4) | 3.0 (1.7, 3.8) | 0.23 |
| Estimated energy intake kcal, median (IQR) | 1955.1 (1795.5, 2080.5) | 2033.0 (1877.2, 2158.4) | 0.27 |
| Reporting error rate %, median (IQR) | -24.5 (-42.5, -22.8) | -18.3 (-40.0, -12.9) | 0.13 |
| Over / Under reporting, n (%) | 9 (64.3) | 9 (64.3) | 1.00 |
| Corrected VD intake μg/day, median (IQR) | 8.2 (4.9, 11.2) | 5.9 (4.2, 8.4) | 0.41 |
| Corrected VD intake ≥ 9 μg/day, n (%) | 7 (50.0) | 3 (21.4) | 0.24 |
| Average VD synthesis μg/day, median (IQR) | 9.7 (8.2, 11.6) | 35.2 (21.9, 42.6) | < 0.001 |
| Average VD synthesis ≥ 10 μg/day, n (%) | 7 (50.0) | 14 (100.0) | 0.006 |
| Serum concentration |  |  | 0 |
| 25(OH)D3 ng/mL, median (IQR) | 13.2 (11.2, 16.9) | 16.5 (12.3, 20.1) | 0.41 |
| Intact-PTH pg/mL, median (IQR) | 30.5 (26.8, 41.8) | 33.5 (27.3, 40.5) | 0.71 |
| Corrected Ca mg/dL, median (IQR) | 9.4 (9.0, 9.5) | 9.3 (9.0, 9.3) | 0.58 |
| P mg/dL, median (IQR) | 3.5 (3.3, 3.7) | 3.7 (3.2, 3.8) | 0.78 |
| PMS, n (%) | 10 (71.4) | 9 (64.3) | 1.00 |
| Physical symptoms, n (%) | 11 (78.6) | 10 (71.4) | 1.00 |
| Mental symptoms, n (%) | 6 (42.9) | 8 (57.1) | 0.71 |
| Sleep-related symptoms, n (%) | 9 (64.3) | 6 (42.9) | 0.45 |
| Use of oral contraceptives, n (%) | 1 (7.1) | 4 (28.6) | 0.33 |
| PHQ-9 score, median (IQR) | 1.5 (1.0, 3.0) | 4.0 (1.0, 6.0) | 0.07 |
| ≥ 5, n (%) | 1 (7.1) | 7 (50.0) | 0.03 |
| AIS score, median (IQR) | 2.0 (1.0, 3.8) | 4.0 (3.0, 5.0) | 0.03 |
| ≥ 6, n (%) | 2 (14.3) | 3 (21.4) | 1.00 |
| Difficulty of initiating sleep, n (%) | 5 (35.7) | 9 (64.3) | 0.26 |
| Difficulty of maintaining sleep, n (%) | 1 (7.1) | 2 (14.3) | 1.00 |
| Early wakening, n %) | 2 (14.3) | 2 (14.3) | 1.00 |
| Daytime sleepiness, n (%) | 8 (57.1) | 12 (85.7) | 0.21 |
| RLS, n (%) | 3 (21.4) | 4 (28.6) | 1.00 |
| Sleep diary |  |  |  |
| Total sleep time (TST) hr, median (IQR) | 6.56 (6.00, 7.00) | 6.00 (5.31, 6.69) | 0.23 |
| < 6 hr, n (%) | 1 (7.1) | 2 (14.3) | 0.76 |
| 6–7 hr. n (%) | 7 (50.0) | 5 (35.7) |  |
| ≥ 7 hr, n (%) | 6 (42.9) | 7 (50.0) |  |
| Range of TST hr, median (IQR) | 4.38 (3.00, 6.00) | 4.00 (2.56, 4.75) | 0.60 |
| Irregularity of TST hr/week, median (IQR) | 6.87 (4.62, 10.45) | 5.44 (4.53, 7.69) | 0.71 |
| Bedtime h:mm, median (IQR) | 1:00 (0:18, 2:00) | 1:15 (0:15, 1:45) | 0.89 |
| Sleep latency min, median (IQR) | 18.8 (0.0, 30.0) | 30.0 (18.8, 30.0) | 0.06 |
| Sleep onset h:mm, median (IQR) | 1:52 (0:45, 2:26) | 1:45 (0:52, 2:41) | 0.80 |
| MS h:mm, median (IQR) | 4:46 (4:03, 5:35) | 5:00 (3:41, 5:46) | 0.95 |
| Irregularity of MS min/week, median (IQR) | 311.25 (204.37, 466.87) | 281.25 (211.87, 336.56) | 0.65 |
| Wake after sleep onset min, median (IQR) | 0.0 (0.0, 0.0) | 0.0 (0.0, 0.0) | 1.00 |
| Sleep offset h:mm, median (IQR) | 8:30 (6:48, 9:00) | 7:52 (7:00, 9:00) | 0.80 |
| Wake-up time h:mm, median (IQR) | 8:33 (7:18, 9:22) | 8:00 (7:33, 9:00) | 0.76 |
| Sleep efficiency %, median (IQR) | 93.1 (89.6, 93.8) | 88.4 (85.8, 92.0) | 0.08 |
| Restorative sleep score, mean (SD) | 69.3 (14.9) | 73.6 (12.9) | 0.43 |
| Daytime dysfunction score, mean (SD) | 30.2 (16.5) | 30.5 (24.0) | 0.96 |
| Percentage breakfast intake %, median (IQR) | 64.3 (44.6, 92.9) | 71.4 (23.2, 78.6) | 0.61 |

AIS, Athens Insomnia Scale; BMI, body mass index; IQR, interquartile range; 25(OH)D, 25-hydroxyvitamin D; MS, midpoint of sleep; PHQ-9, Patient Health Questionnaire-9; PMS, premenstrual syndrome; PTH, parathyroid hormone; RLS, restless legs syndrome; SD, standard deviation; TST, total sleep time; VD, vitamin D.

Supplementary Figure 4. Correlation matrix and scatterplot matrix for summer AIS scores, PHQ-9 scores, serum 25(OH)D concentrations, vitamin D synthesis amounts, and vitamin D intake amounts. The upper right shows the correlation matrix, the lower left shows the scatterplot matrix, and the diagonal displays density plots for each variable. The correlation matrix presents Spearman's *ρ* and *P* values. For scatterplots where significant correlations were observed, the least squares regression line is also shown. AIS, Athens Insomnia Scale; 25(OH)D, serum 25-hydroxyvitamin D; PHQ-9, Patient Health Questionnaire-9; VD. vitamin D.
